# Adaptive parameter estimation for the expanded sandwich model

**DOI:** 10.1038/s41598-023-36888-6

**Published:** 2023-06-16

**Authors:** Guanglu Yang, Huanlong Zhang, Yubao Liu, Qingling Sun, Jianwei Qiao

**Affiliations:** 1 Nanyang Cigarette Factory of Henan China Tobacco Industry Co., Ltd, Nanyang, 473000 People’s Republic of China; 2 College of Electrical and Information Engineering, Zhengzhou University of Light Industry, Zhengzhou, 450000 People’s Republic of China; 3 Wolong Electric Nanyang explosion proof motor Group Co., Ltd, Nanyang, 473000 People’s Republic of China

**Keywords:** Mechanical engineering, Computational science

## Abstract

An expanded-sandwich system is a nonlinear extended block-oriented system in which memoryless elements in conventional block-oriented systems are displaced by memory submodels. Expanded-sandwich system identification has received extensive attention in recent years due to the powerful ability of these systems to describe actual industrial systems. This study proposes a novel recursive identification algorithm for an expanded-sandwich system, in which an estimator is developed on the basis of parameter identification error data rather than the traditional prediction error output information. In this scheme, a filter is introduced to extract the available system information based on miserly structure layout, and some intermediate variables are designed using filtered vectors. According to the developed intermediate variables, the parameter identification error data can be obtained. Thereafter, an adaptive estimator is established by integrating the identification error data compared with the classic adaptive estimator based on the prediction error output information. Thus, the design framework introduced in this research provides a new perspective for the design of identification algorithms. Under a general continuous excitation condition, the parameter estimation values can converge to the true values. Finally, experimental results and illustrative examples indicate the availability and usefulness of the proposed method.

## Introduction

**Figure 1:**
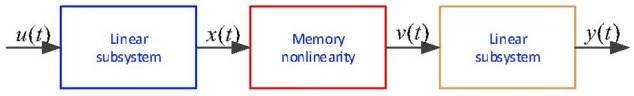
Extended sandwich model.

In recent decades, although linear models that can describe the characteristics of an actual system have been developed, the ability of these systems to describe such a system with inherent nonlinear characteristics has been limited or even a failure^1–3^. Consequently, a variety of nonlinear models have been used to establish mathematical dynamic models for practice systems according to the requirements of users. Additionally, nonlinear models provide stronger representation abilities than linear models due to their nonlinear submodels. The block-oriented model (BOM) is one of the nonlinear models, including nonlinear sub-models^4–6^. By selecting different linear subsystems and nonlinear models, the BOM can describe the inherent characteristics of numerous actual systems. The traditional BOM uses memoryless elements to enhance the description ability of the model, but it is not ideal for an actual system with memory nonlinear characteristics. To solve the preceding problem, so-called expanded block-oriented models have been proposed by displacing memoryless elements based on memory nonlinear sub-models^7,8^. Among the extended BOMs, the extended sandwich model shown in Fig. 1 is a popular model because of its unique structure. Moreover, the extended sandwich model can establish effective mathematical models for numerous systems, such as stirred tank reactor systems^9^, optical transmitters^10^, medical surgical systems^11^, and servo systems^12^, etc. Thus, discussion of extended sandwich system identification method is beneficial to intuitively understand the modeling processes of actual systems and the presentation forms of inherent nonlinear characteristics.

Effective and novel identification schemes for the extended BOMs have been reported^7,13,14^. The majority of existing reports on extended BOM identification have mainly focused on expanded Hammerstein and expanded Wiener systems. Only a few published works have been conducted on the expanded Hammerstein-Wiener and Wiener-Hammerstein systems because these two systems are markedly challenging to system identification^15–18^. In the convergence performance aspect, Li^19^ proposed an improved multi-innovation gradient method for parameter estimations of the extended sandwich system, in which the multi-innovation length is modified to increase the data utilization rate, thereby enhancing the convergence rate. A least-squares method based on internal iteration was introduced as Vörös in^20^, in which the internal iteration idea produces a rapid convergence performance. In^21^, Quaranta discussed the identification of an extended sandwich system with hysteresis nonlinearity by developing intelligent optimization algorithm. An adaptive identification scheme was investigated based on guaranteed performance, to reduce the convergence time. Additionally, a method with improved performance was proposed in^22^. Zhou *et al*.^12^ used a nonsmooth Kalman filter based on the nonsmooth stochastic state-space equation to address noise signal, and to increase estimation accuracy. The preceding estimation methods can effectively achieve system identification for the extended BOMs. However, the adaptive law is mostly developed with prediction error output or observation error data because the identification regression form is easy to obtain. When noise intensity is slightly high or the estimation model is complex, prediction error data will produce biased estimation and minimum problems. To avoid this deficiency, we search for other error data to develop an adaptive law, which is the motivation of the current research. Note that the adaptive parameter estimation law is modified and updated according to the effective error data. If the adaptive law can be modified by the parameter estimation error, which is directly related to the parameter estimation process, then the estimation performance will be substantially improved. Therefore, we use parameter identification error data to derive an alternative adaptive law.

Noise coexists with system data during the process of collecting identification data. Several filters for reducing noise signals have been proposed^23–27^. A linear filter was used to obtain filtered input and output information, and an overparameterization scheme was proposed to recover parameter information in^28^. Ding^29^ reported an adaptive Kalman filter for nonlinear systems, in which the parameter and state could be effectively estimated. To decrease student-t-distributed noise, Wang proposed a robust filter to improve the estimation accuracy, and derived the Cramer-Rao bounds thereafter^30^. A diffusion particle filter was introduced by de Figueredo^31^ to identify parameters of the unit sphere based on a network, in which the proposed algorithm outperformed the Kalman filter method. Subudhi used $H_{\infty }$ filter on the basis of a sparse model, and the error convergence accuracy of the identification model was improved^32^. The majority of the reported filters in the published papers can implement effective estimation under several assumptions. In applications, some of these assumptions are strict. Relaxing the filter assumption is an open topic, which also satisfies the requirements of practical applications. Accordingly, we propose a filter operator to obtain the beneficial identification data from contaminated system data.

Inspired by the related works, a novel recursive identification approach for an expanded sandwich systems is introduced. The main contributions of the paper are listed as follows:

1. The introduced filter possesses a simple structure and relaxed assumptions about the considered system compared to those of some filters^23–25^.
2. An estimation error extraction method is given based on some filtered matrices and vectors, this approach is different from the commonly used error construction method.
3. A novel parameter estimation law is yielded by integrating the estimation error instead of the common prediction error output or observation error data^7,13–19^.

The remainder of this study is summarized as follows. In the next section, a brief summary of the system description is stated. The developed method is introduced in "Adaptive identification scheme" section. The theoretical analysis is described in “Convergence analysis” section. In Example verification and experiment section, examples are provided. The conclusion of this study is offered in the last section.

## Problem statement

The expanded sandwich system shown in Fig. 1 can be described mathematically as follows:

The first linear subsystem:

1 $$\begin{aligned} B(q^{-1})x(t)=A(q^{-1})u(t), \end{aligned}$$

The memory nonlinear submodel:

2 $$\begin{aligned} v(t)= & {} {\left\{ \begin{array}{ll} k_{l}(x(t)+b_{l})&{} if\;x(t)< x_{l}\\ v(t-1) &{} if\;x_{l}\le x(t)\le x_{r}\\ k_{r}(x(t)-b_{r}) &{}if\;x(t)< x_{r},\\ \end{array}\right. } \end{aligned}$$

3 $$\begin{aligned} x_{l}= & {} v(t)/k_{l}-b_{l}, x_{r}=v(t)/k_{r}+b_{r}, \end{aligned}$$

The second linear subsystem:

4 $$\begin{aligned} D(q^{-1})y(t)=C(q^{-1})v(t)+e(t), \end{aligned}$$

where $A(q^{-1})$, $B(q^{-1})$, $C(q^{-1})$ and $D(q^{-1})$ are polynomial with *q*. System input-output sequence is described by $\{u(t),y(t)\}$, the internal signals are denoted by *v*(*t*) and *x*(*t*), respectively. *e*(*t*) is an addition noise sequence. $k_{l}$ and $k_{r}$ are two slopes, $b_{l}$ and $b_{r}$ be the intersections with the signal *x*(*t*) axis. $q^{-1}$ be unit delay operator with $q^{-1}x(t)=x(t-1)$, $A(q^{-1})$, $B(q^{-1})$, $C(q^{-1})$ and $D(q^{-1})$ are given by

5 $$\begin{aligned} \left\{ \begin{aligned} A(q^{-1})&=a_{1}q^{-1}+a_{2}q^{-2}+\cdots +a_{m}q^{-m}, \\ B(q^{-1})&=1+b_{1}q^{-1}+\cdots +b_{n}q^{-n},\\ C(q^{-1})&=c_{1}q^{-1}+c_{2}q^{-2}+\cdots +c_{s}q^{-z},\\ D(q^{-1})&=1+d_{1}q^{-1}+\cdots +d_{w}q^{-w}. \end{aligned} \right. \end{aligned}$$

### Assumption 1

The two linear subsystems are stable.

### Assumption 2

The limited degrees *m*, *n*, *z*, *w* are set by user, the constants $a_{i}$, $b_{j}$, $c_{j}$,$d_{i}$ are unknown.

### Assumption 3

The addition noise and input signal are independent.

### Assumption 4

The initial states of the system are assumed to be zero.

### Assumption 5

The system can be fully excited by selecting the input signal.

### Assumption 6

The constants $a_{1}=1,c_{1}=1$ are set.

The working conditions of linear subsystems are shown in Assumption 1. Assumption 2 displays the system order information and the estimated parameter information. The noise assumption condition is described in Assumption 3. Assumption 4 indicates that the considered system is memoryless before identification data are collected. Assumption 5 shows the basic condition for system identifiability. In Assumption 6, a model uniqueness condition is provided^33^.

As shown in Eq. (2), the memory block has backlash nonlinearity. The backlash characteristic widely exists in various pieces of mechanical equipment due to the presence of gears^34,35^. Hence, we use the backlash submodel to represent memory nonlinearity. The linear expression of backlash nonlinearity can be defined as in^36,37^

6 $$\begin{aligned} \begin{aligned} v(t)=&k_{l}x(t)g_{1}(t)+k_{l}b_{l}g_{1}(t)+k_{r}x(t)g_{2}(t)-k_{r}b_{r}g_{2}(t)\\&+v(t-1)g_{1}(t)g_{2}(t)+v(t-1)-g_{1}(t)v(t-1)\\&-g_{2}(t)v(t-1), \end{aligned} \end{aligned}$$

where

7 $$\begin{aligned}{} & {} \left\{ \begin{aligned} g_{1}(t)&=R[x(t)-x_{l}] \\ g_{2}(t)&=R[x_{r}-x(t)], \end{aligned} \right. \end{aligned}$$

8 $$\begin{aligned}{} & {} R(t)= {\left\{ \begin{array}{ll} 0&{}\quad t > 0,\\ 1&{}\quad t\le 0,\\ \end{array}\right. } \end{aligned}$$

where $g_{1}(t)$ and $g_{2}(t)$ are used to describe the three branching mapping conditions, *R*(*t*) denotes a switching function.

Based on (1), (3) and (6), the compact formal identification model is described as

9 $$\begin{aligned} y(t)=\Theta ^T\xi (t)+e(t), \end{aligned}$$

where the observation data is provided by

$$\xi (t)=[g_{1}(t-1)u(t-2),\cdots ,g_{1}(t-1)u(t-m-1),-g_{1}(t-1)x(t-2), \cdots ,-g_{1}(t-1)x(t-n-1) ,g_{1}(t-1),g_{2}(t-1)x(t-1),-g_{2}(t-1),v(t-2)[1-g_{1}(t-1)][1-g_{2}(t-1)],v(t-2),\cdots ,v(t-z),-y(t-1),\cdots ,-y(t-w)]^{T}$$,

and the estimated parameter variable is written as

$$\Theta =[k_{l}c_{1}a_{1},\cdots , k_{l}c_{1}a_{m}, k_{l}c_{1}b_{1},\cdots , k_{l}c_{1}b_{n}, k_{l}c_{1}b_{l},k_{r}c_{1}, k_{r}b_{r}c_{1},c_{1},\cdots ,c_{z}, d_{1},\cdots ,d_{w}]^{T}$$.

### Remark 1

According to Assumption 6, $\Theta$ is transformed into $\Theta =[k_{l},\cdots , k_{l}a_{m}, k_{l}b_{1},\cdots , k_{l}b_{n}, k_{l}b_{l},k_{r},k_{r}b_{r},1, \cdots ,c_{z}, d_{1},\cdots ,d_{w}]^{T}$. By using simple mathematical operations, the each estimated parameter can be obtained.

This research aims to develop an adaptive recursive identification method for an expanded sandwich system, investigate the convergence performance of the method from a theory perspective, and examine the efficiency of the developed method by using some examples to compare it with the existing identification methods.

## Adaptive identification scheme

**Figure 2:**
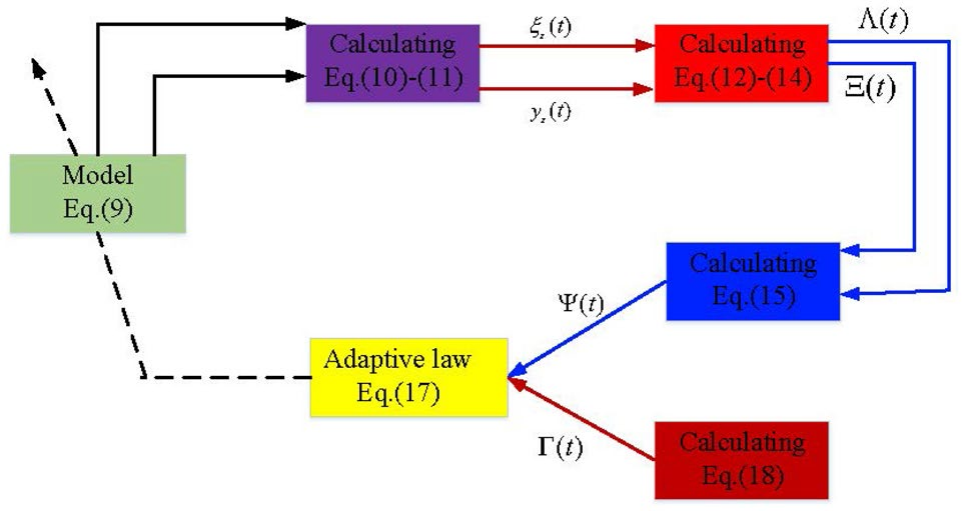
Flow chart of developed method.

This section introduces a recursive estimation approach for the system considered in “Problem statement” section, and compared with the classic recursive method, this paper provides an alternative estimation algorithm design. To ensure the integrity of the paper, Fig. 2 shows the flow chart of the developed method. First, a filter operator is introduced to yield the filtered identification information. Second, on the basis of the introduced filtered variables, identification error information is obtained. Finally, by using the error information of the parameter identification process, a new adaptive law for parameter estimation can be developed, wherein the structure of a novel estimation method is given by using parameter error information rather than the popularly utilized prediction error output information.

A filter operator is introduced to relieve the above assumption and restrain the influence of noise. For this reason, observation and output data need to be filtered. Meanwhile, defining the filtered data $y_{\epsilon }(t)$ and $\xi _{\epsilon }(t)$, it yields

10 $$\begin{aligned} y_{\epsilon }(t)= & {} \frac{\alpha }{\alpha +1}y_{\epsilon }(t-1)+\frac{1}{\alpha +1}y(t), \end{aligned}$$

11 $$\begin{aligned} \xi _{\epsilon }(t)= & {} \frac{\alpha }{\alpha +1}\xi _{\epsilon }(t-1)+\frac{1}{\alpha +1}\xi (t), \end{aligned}$$

where the constant $\alpha$ with simple form describes the filter operator. $y_{\epsilon }(0)=0.001$, $\xi _{\epsilon }(0)=0.001$.

To avoid the weakness of the prediction error output or observation error data, we use the estimation error data to develop a new adaptive law. To this end, we need to introduce a method for extracting estimation error data from the observed system data. By defining the intermediate variables $\Lambda (t)$ and $\Xi (t)$, we have

12 $$\begin{aligned} \Lambda (t)= & {} \frac{1}{1+\gamma (t)}\Lambda (t-1)+\frac{1}{1+\gamma (t)}\xi _{\epsilon }(t)\xi _{\epsilon }^T(t), \end{aligned}$$

13 $$\begin{aligned} \Xi (t)\!= & {} \! \frac{1}{1+\gamma (t)}y_{\epsilon }(t)\xi _{\epsilon }^{-1}(t)\Lambda (t-1)\!+\!\frac{ y_{\epsilon }(t)\xi _{\epsilon }^T(t)}{1+\gamma (t)}, \end{aligned}$$

14 $$\begin{aligned} \gamma (t)= & {} e^{-\tau t}/(1+e^{-\tau t})^2,\tau >0, \end{aligned}$$

where the forgetting coefficient is denoted by $\gamma (t)$. $\Lambda (0)=0.001$, $\Xi (0)=0.001$.

### Remark 2

The filter operator $\alpha$ with miserly form can obtain filtered data, thereby simplifying the filter design. The forgetting coefficient $\gamma (t)$ improves the availability of identification data, to avoid the so-called data flooding phenomenon and enhance the convergence rate of the method.

Based on (12)–(13), the auxiliary variable $\Psi (t)$ is defined by using the following form

15 $$\begin{aligned} \Psi (t)={\hat{\Theta }}^T(t-1)\Lambda (t)-\Xi (t), \end{aligned}$$

where ${\hat{\Theta }}(t)$ denotes the estimated value of $\Theta (t)$.

Define the identification error ${\tilde{\Theta }}(t-1)$, ${\tilde{\Theta }}(t-1)=\Theta -{\hat{\Theta }}(t-1)$, (15) can be rewritten from (12)–(13) as follows

16 $$\begin{aligned} \Psi (t)=-{\tilde{\Theta }}^T(t-1)\Lambda (t)+\varepsilon (t), \end{aligned}$$

where $\varepsilon (t)=-e_{\epsilon }(t)\xi _{\epsilon }^T(t)/(1+\gamma (t))$, $e_{\epsilon }(t)$ is filtered variable of *e*(*t*).

### Remark 3

The majority of adaptive parameter laws are induced based on the prediction error output or observation error data. The reason for this is that the accessibility of these two types of error data, which leads to an adaptive update law, is corrected by using information indirectly that is related to the parameter error. When the parameter estimation error is used to modify the adaptive law, the parameter estimation process achieves superior performance because the estimation error is directly related to the parameter estimation. This result is consistent with the principle of using feedback error data to correct the actual error.

As stated in Remark 3, the estimation error data can enhance the identification behaviour. Thus, the following adaptive law is written

17 $$\begin{aligned} {\hat{\Theta }}(t)={\hat{\Theta }}(t-1)-\Gamma (t)\Lambda (t)\Psi ^T(t). \end{aligned}$$

To achieve the operability of online implementation, the modified gain $\Gamma (t)$ with recursive form is designed. Based on the system data $\Lambda (t)$, the expression of $\Gamma (t)$ is given as

18 $$\begin{aligned} \begin{aligned} \Gamma (t)=\Gamma (t-1)-\frac{\Gamma (t-1)\Lambda (t)\Lambda ^T(t) \Gamma (t-1)}{E+\Lambda ^T(t)\Gamma (t-1)\Lambda (t)}, \end{aligned} \end{aligned}$$

where *E* represents unit matrix with appropriate dimension.

### Remark 4

From (16), we define $\Psi (t)$ as an extended identification error variable because the estimation error ${\hat{\Theta }}(t-1)$ is integrated into $\Psi (t)$. Thereafter, the identification error variable is used to construct an adaptive update law, in which a new perspective for designing an estimation method by using parameter error data is shown and compared with the classic identification scheme. The recursive modified gain $\Gamma (t)$ improves the efficiency of the online operation, and the speed of the parameter update process in comparison with that of the common constant gain.

**Figure 3:**
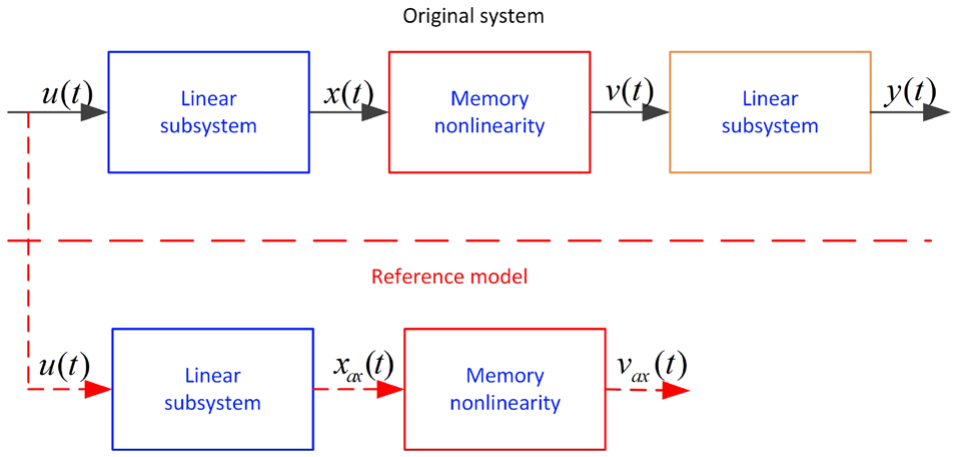
Expanded sandwich system with reference model.

It can be observed from Fig. 1, *x*(*t*) and *v*(*t*) are immeasurable. We need to address these unmeasured variables to obtain an effective parameter estimation using the developed method. One solution based on the original system is to design reference models^38–40^ specifically by using the reference model output data to substitute for the unmeasured *x*(*t*) and *v*(*t*), as shown in Fig. 3. Thereafter, the reference models of $x_{ax}(t)$ and $v_{ax}(t)$ are described as follows

19 $$\begin{aligned}{} & {} \begin{aligned} x_{ax}(t)&=-{\hat{b}}_{1}x_{ax}(t-1)-,\cdots ,-{\hat{b}}_{n}x_{ax}(t-n)+{\hat{a}}_{1}u(t-1)\\&+,\cdots ,+{\hat{a}}_{m}u(t-m), \end{aligned} \end{aligned}$$

20 $$\begin{aligned}{} & {} \begin{aligned} v_{ax}(t)=&\hat{k_{l}}x_{ax}(t)g_{1}(t)+\widehat{k_{l}b_{l}}g_{1}(t)+\hat{k_{r}}x_{ax}(t)g_{2}(t)-\widehat{k_{r}b_{r}}g_{2}(t) \\&+v_{ax}(t-1)g_{1}(t)g_{2}(t)+v_{ax}(t-1)-g_{1}(t)v_{ax}(t-1)\\&-g_{2}(t)v_{ax}(t-1). \end{aligned} \end{aligned}$$

Next, the convergence of the developed method is introduced from theoretical analysis perspective.

## Convergence analysis

This section will introduce the convergence analysis of the proposed estimation approach. Firstly, we establish an extended Lyapunov function based on error data. Secondly, we use the martingale difference convergence theorem and scaling principle to gradually deduce the estimation error expression. Lastly, when the time approaches infinity, it is verified whether or not the estimation error approaches zero.

### Theorem 1

It is assumed that $\{\varepsilon (t),{\mathscr {F}}_{t}\}$ is martingale difference sequence, $\{{\mathscr {F}}_{t}\}$ is produced by using the observation data when $0\le t' \le t$. $\varepsilon (t)$ satisfies the conditions^41^

(F1) $$E[\varepsilon (t)|{\mathscr {F}}_{t-1}]=0,$$

(F2) $$E[\varepsilon ^2(t)|{\mathscr {F}}_{t-1}]\le \sigma _{\varepsilon }^2<\infty $$,

(F3) $$\alpha _{0} I_{n}\le 1/t\sum _{i=1}^{t}\Lambda (i)\Lambda ^T(i)\le \alpha _{1} I_{n}$$, $$\alpha _{0}>0, \alpha _{1}>0$$

Then, the error obtained by the proposed method converges to zero, i.e.,

$$\begin{aligned} \lim \limits _{t\rightarrow \infty }\Vert {\tilde{\Theta }}(t)\Vert =\Vert {\hat{\Theta }}(t)-\Theta \Vert ^2=0 \end{aligned}$$

### Proof

By subtracting $\Theta$ at both ends of (17), it obtains

21 $$\begin{aligned} \begin{aligned} {\tilde{\Theta }}(t)&={\tilde{\Theta }}(t-1)+\Gamma (t)\Lambda (t)[-{\tilde{\Lambda }}(t)+\varepsilon (t)], \end{aligned} \end{aligned}$$

where ${\tilde{\Lambda }}(t)$ is defined by ${\tilde{\Lambda }}(t)={\tilde{\Theta }}^T(t-1)\Lambda (t)$.

To analyse the convergence of estimation error, define $X(t)={\tilde{\Lambda }}^T(t)\Gamma ^{-1}(t){\tilde{\Lambda }}(t)$, by substituting (21) into *X*(*t*), it yields

22 $$\begin{aligned} \begin{aligned} X(t)=&[{\tilde{\Theta }}(t-1)+\!\Gamma (t)\Lambda (t)(-{\tilde{\Lambda }}(t)\!+\!\varepsilon (t))]^T\Gamma ^{-1}(t)\\&\times [{\tilde{\Theta }}(t-1)+\Gamma (t)\Lambda (t)(-{\tilde{\Lambda }}(t)+\varepsilon (t))]\\ =&{\tilde{\Theta }}^T(t-1)\Gamma ^{-1}(t){\tilde{\Theta }}(t-1)\!+\!2{\tilde{\Theta }}^T(t-1)\Lambda (t)\\&\times (-{\tilde{\Lambda }}(t)\!+\!\varepsilon (t))\!+\!\Lambda ^T(t)\Gamma (t)\Lambda (t)(-{\tilde{\Lambda }}(t)\!+\!\varepsilon (t))^2\\ =&X(t-1)-{\tilde{\Lambda }}^2(t)+2{\tilde{\Lambda }}(t)\varepsilon (t)\!+\!\Lambda ^T(t)\Gamma (t)\\&\times \Lambda (t) {\tilde{\Lambda }}^2(t)+\Lambda ^T(t)\Gamma (t)\Lambda (t)\varepsilon ^2(t)\\&-2\Lambda ^T(t)\Gamma (t)\Lambda (t){\tilde{\Lambda }}(t)\varepsilon (t)\\ =&X(t-1)-[1-\Lambda ^T(t)\Gamma (t)\Lambda (t)]{\tilde{\Lambda }}^2(t)\\&+2[1-\Lambda ^T(t)\Gamma (t)\Lambda (t)]{\tilde{\Lambda }}(t)\varepsilon (t)+\Lambda ^T(t)\Gamma (t)\\&\times \Lambda (t)\varepsilon ^2(t). \end{aligned} \end{aligned}$$

By applying matrix inversion theory to (18), the following inequality holds

23 $$\begin{aligned} \begin{aligned} 1-\Lambda ^T(t)\Gamma (t)\Lambda (t)=&1-\Lambda ^T(t)[\Gamma (t-1)\\&-\frac{\Gamma (t-1)\Lambda (t)\Lambda ^T(t)\Gamma (t-1)}{E+\Lambda ^T(t)\Gamma (t-1)\Lambda (t)}]\\&\times \Lambda (t)\\ =&1-\frac{\Lambda ^T(t)\Gamma (t-1)\Lambda (t)}{E+\Lambda ^T(t)\Gamma (t-1)\Lambda (t)}]\\ =&\frac{E}{E+\Lambda ^T(t)\Gamma (t-1)\Lambda (t)}>0. \end{aligned} \end{aligned}$$

According to (23), (22) has

24 $$\begin{aligned} \begin{aligned} X(t)\le&X(t-1)+\Lambda ^T(t)\Gamma (t)\Lambda (t)\varepsilon ^2(t)\\&+2[1-\Lambda ^T(t)\Gamma (t)\Lambda (t)]{\tilde{\Lambda }}(t)\varepsilon (t). \end{aligned} \end{aligned}$$

By using the martingale convergence theorem to (24) and combining (F1)–(F2), the following expression is derived

25 $$\begin{aligned} E[X(t)|{\mathscr {F}}_{t-1}]\le X(t-1)+2 \Lambda ^T(t)\Gamma (t)\Lambda (t)\sigma ^2_{\varepsilon }, \end{aligned}$$

where the conditional expectation is described by $E(\cdot \mid \cdot )$.

Continuing with the following derivation, define $H(t)=\frac{X(t)}{[\ln |\Gamma ^{-1}(t)|]^{\rho }}, \rho >1$, it yields

26 $$\begin{aligned} \begin{aligned} E[H(t)|{\mathscr {F}}_{t-1}]&\le \frac{X(t-1)}{[\ln |\Gamma ^{-1}(t)|]^{\rho }}+\frac{\Lambda ^T(t)\Gamma (t)\Lambda (t) \sigma ^2_{\varepsilon }}{[\ln |\Gamma ^{-1}(t)|]^{\rho }}\\&\le H(t-1)+\frac{\Lambda ^T(t)\Gamma (t)\Lambda (t)\sigma ^2_{\varepsilon }}{[\ln |\Gamma ^{-1}(t)|]^{\rho }}. \end{aligned} \end{aligned}$$

Based on martingale theorem, *H*(*t*) has the following expression

27 $$\begin{aligned} H(t)=\frac{X(t)}{[\ln |\Gamma ^{-1}(t)|]^{\rho }}\rightarrow H_{0}<\infty , a.s. \end{aligned}$$

where the finite random variable is denoted by $H_{0}$.

(27) can be rewritten as

28 $$\begin{aligned} X(t)\le \kappa [\ln |\Gamma ^{-1}(t)|]^{\rho } ,a.s., t\rightarrow \infty , \end{aligned}$$

where the large variable is given as $\kappa$.

By using the definition of *X*(*t*), ${\tilde{\Theta }}(t)$ has

29 $$\begin{aligned} \Vert {\tilde{\Theta }}(t)\Vert ^2\le \frac{\kappa [\ln |\Gamma ^{-1}(t)|]^{\rho }}{\lambda _{min} [\Gamma ^{-1}(t)]}\le \frac{\kappa [\ln |\textrm{tr}(\Gamma ^{-1}(t))|]^{\rho }}{\lambda _{min}[\Gamma ^{-1}(t)]}, \end{aligned}$$

where the minimum eigenvalue is denoted by $\lambda _{min}[\cdot ]$, the matrix trace is described by $\textrm{tr}(\cdot )$.

By using (F3) and (18), the following inequalities hold

30 $$\begin{aligned}{} & {} \textrm{tr}(\Gamma ^{-1}(t))\le n\alpha _{1} t+n\Gamma ^{-1}(0), \end{aligned}$$

31 $$\begin{aligned}{} & {} \lambda _{min}[\Gamma ^{-1}(t)]\ge \alpha _{0} t, \end{aligned}$$

where $\Gamma ^{-1}(0)$ describes a finite initial value.

By substituting (30)–(31) into (29), it obtains

32 $$\begin{aligned} \lim \limits _{t\rightarrow \infty }\Vert {\hat{\Theta }}(t)-\Theta \Vert ^2\le \lim \limits _{t\rightarrow \infty }\frac{\kappa [n\ln (n\alpha _{1} t+n\Gamma ^{-1}(0))]^\rho }{\alpha _{0} t}=0, a.s.. \end{aligned}$$

$$\square $$

The proof of Theorem 1 is finished.

## Example verification and experiment

This section applies the considered identification schemes to estimate the extended sandwich system. The comparison methods in this paper are chosen based on the prediction error method because such approach methods (e.g, least square type and gradient type) are the most widely used identification schemes in system identification community. As stated in the introduction, the purpose of this paper is to design an alternative identification algorithm to improve upon the shortcomings of prediction error methods. Hence, we choose the identification algorithms based on the prediction error method as the comparison schemes.

### Illustrative example

The extended sandwich system is listed as follows:

The first linear subsystem:

$$\begin{aligned} (1+b_{1}q^{-1}+b_{2}q^{-2})x(t)=(a_{1}q^{-1}+a_{2}q^{-2})u(t) \end{aligned}$$

The backlash nonlinear submodel:

$$\begin{aligned} v(t)= {\left\{ \begin{array}{ll} k_{l}(x(t)+b_{l})&{} if\;x(t)< x_{l}\\ v(t-1) &{} if\;x_{l}\le x(t)\le x_{r}\\ k_{r}(x(t)-b_{r}) &{}if\;x(t)< x_{r}\\ \end{array}\right. } \end{aligned}$$

The second linear subsystem:

33 $$\begin{aligned} (1+d_{1}q^{-1}+d_{2}q^{-2})y(t)=(c_{1}q^{-1}+c_{2}q^{-2})v(t)+e(t) \end{aligned}$$

where the expected values of the above system parameter are $a_{1}=1$, $a_{2}=0.35$, $b_{1}=0.5$, $b_{2}=0.45$, $k_{l}=k_{r}=0.8$, $b_{l}=b_{r}=0.2$, $c_{1}=1$, $c_{2}=0.1$, $d_{1}=0.4$, $d_{2}=0.3$. In this paper, we propose a recursive identification framework to obtain the parameter information.

The considered system is excited using a random signal with zero mean and unit variable. The system data are contaminated by using a white noise with zero mean and finite variable. The multi-innovation stochastic gradient (MI-SG) in^39^ and the extended recursive identification algorithm (E-RIA)^42^ are chosen as two comparison methods.

To guarantee the parameter estimation implementation process, the initial parameters of the considered estimation methods are provided.

1. Proposed method: $$\alpha =2$$, $$\gamma (0)=0.95$$, $$\tau =3$$, $$\Gamma (0)=200*diag([0.01,0.01,0.01,0.01,0.01,0.01,0.01,0.01])^T, {\hat{\theta }}(0)=I/p0,p0=10^3$$, $$x_{ax}(0)=0.001$$, $$v_{ax}(0)=0.001$$, $$N=800$$.
2. E-RIA: $${\hat{\theta }}(0)=I/p0, p0=10^3$$, $$x_{ax}(0)=0.001$$, $$v_{ax}(0)=0.001$$, $$N=800$$, $$\mu (0)=0.9$$,$$\rho (0)=0.95$$.
3. MI-SG: $${\hat{\Theta }}(0)=I/p0, p0=10^3$$, $$r=1$$, $$x_{ax}(0)=0.001$$, $$p=6$$, $$v_{ax}(0)=0.001$$, $$N=800$$

**Figure 4:**
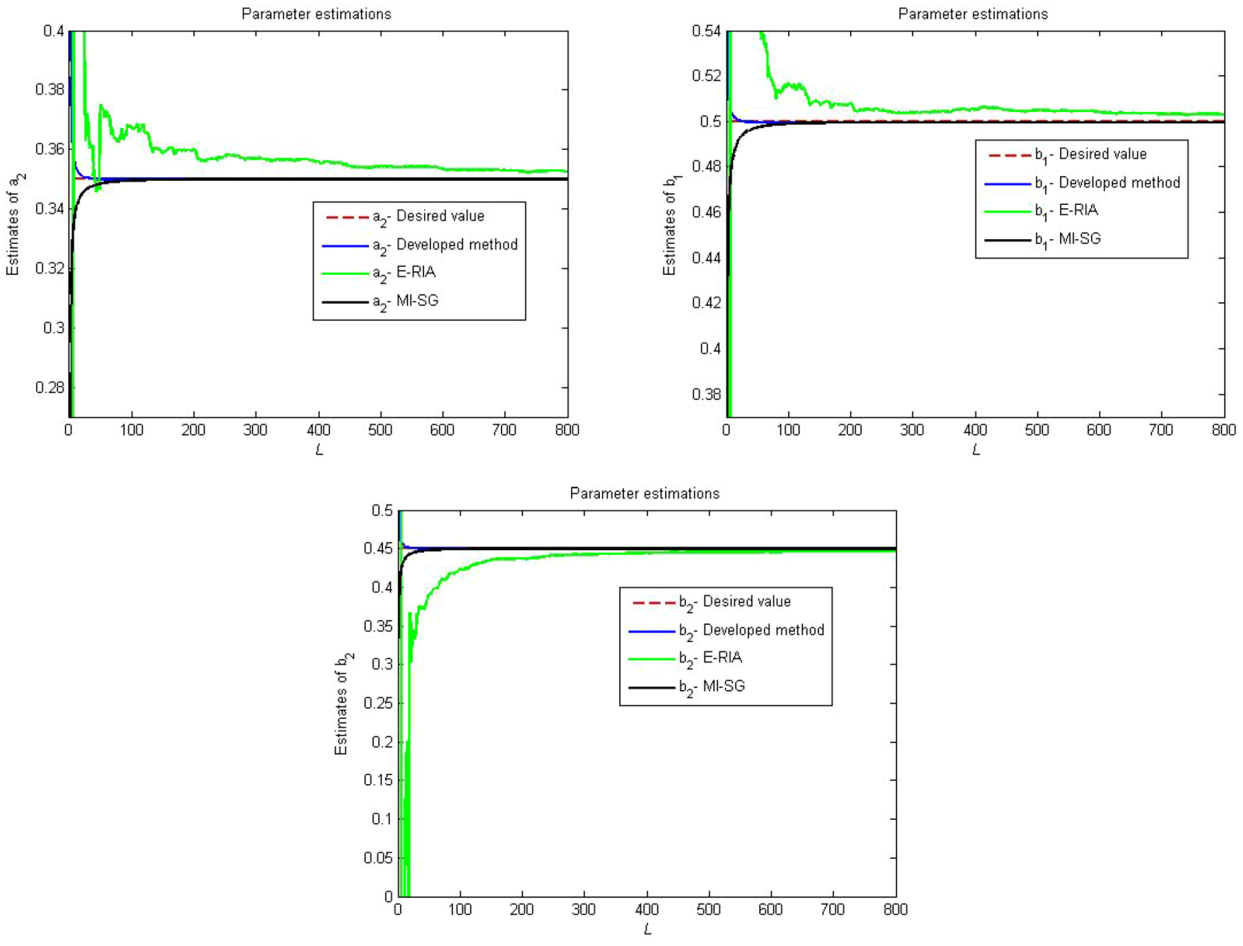
Comparison parameter estimations for first linear subsystem.

**Figure 5:**
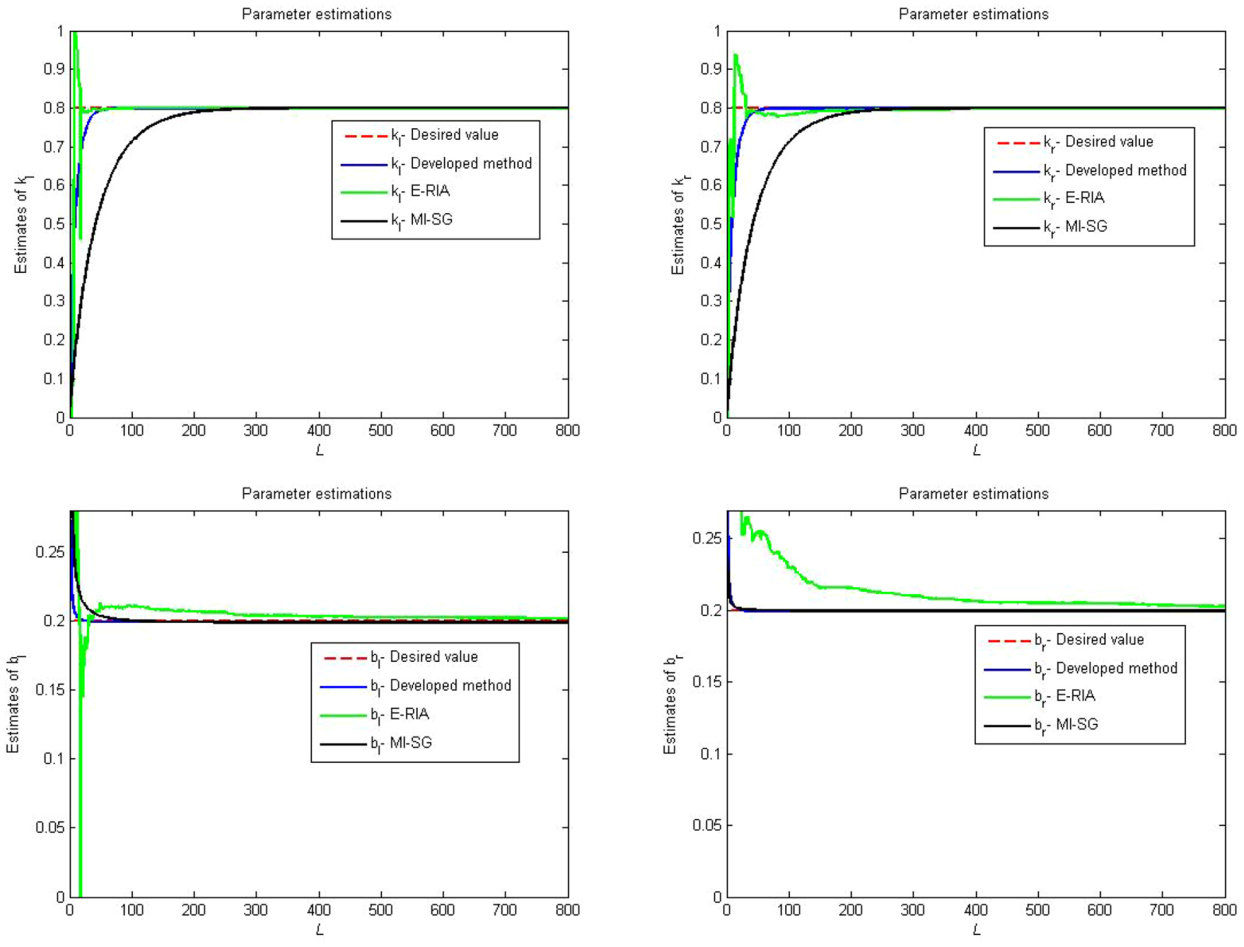
Comparison parameter estimations for backlash.

**Figure 6:**
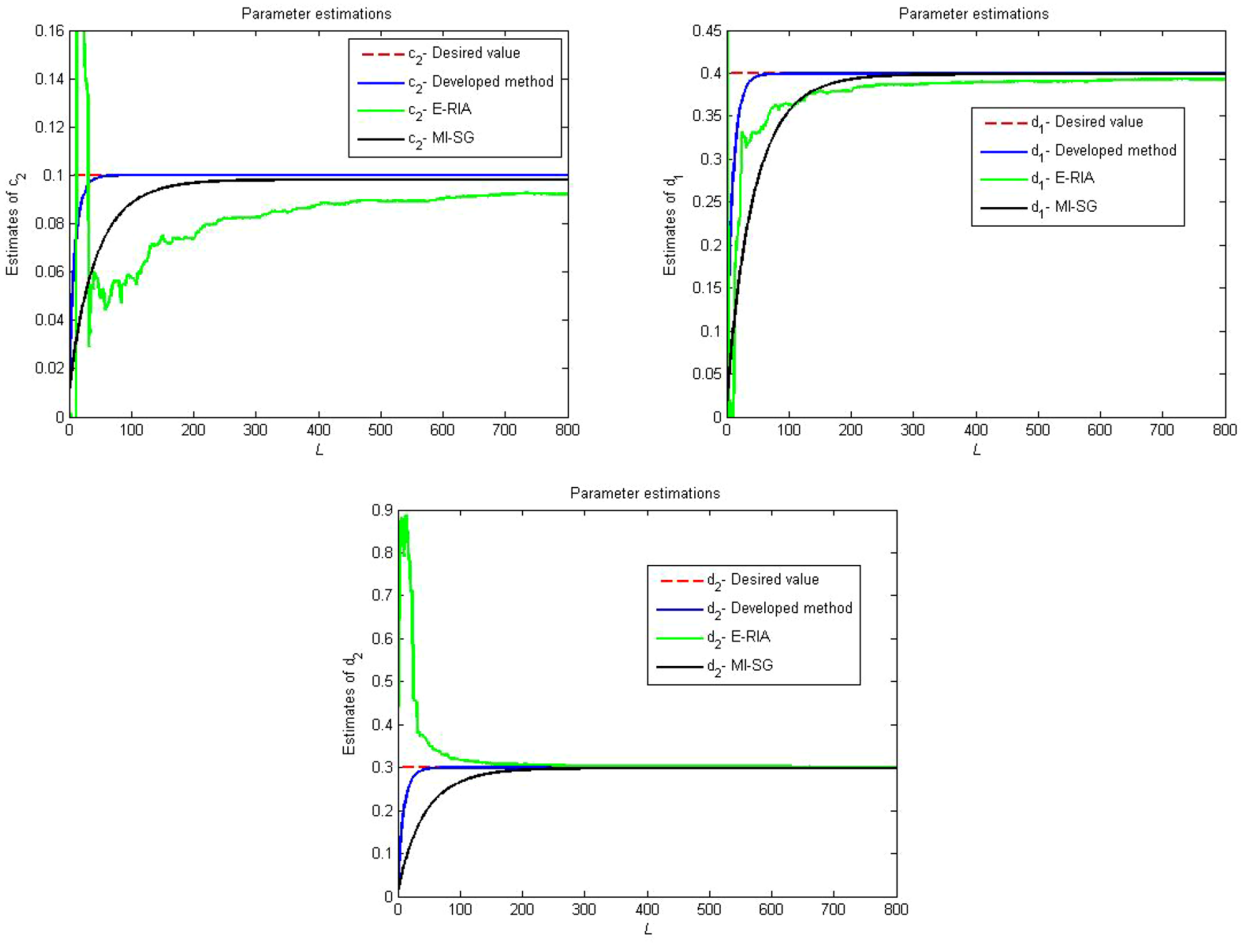
Comparison parameter estimations for second linear subsystem.

**Figure 7:**
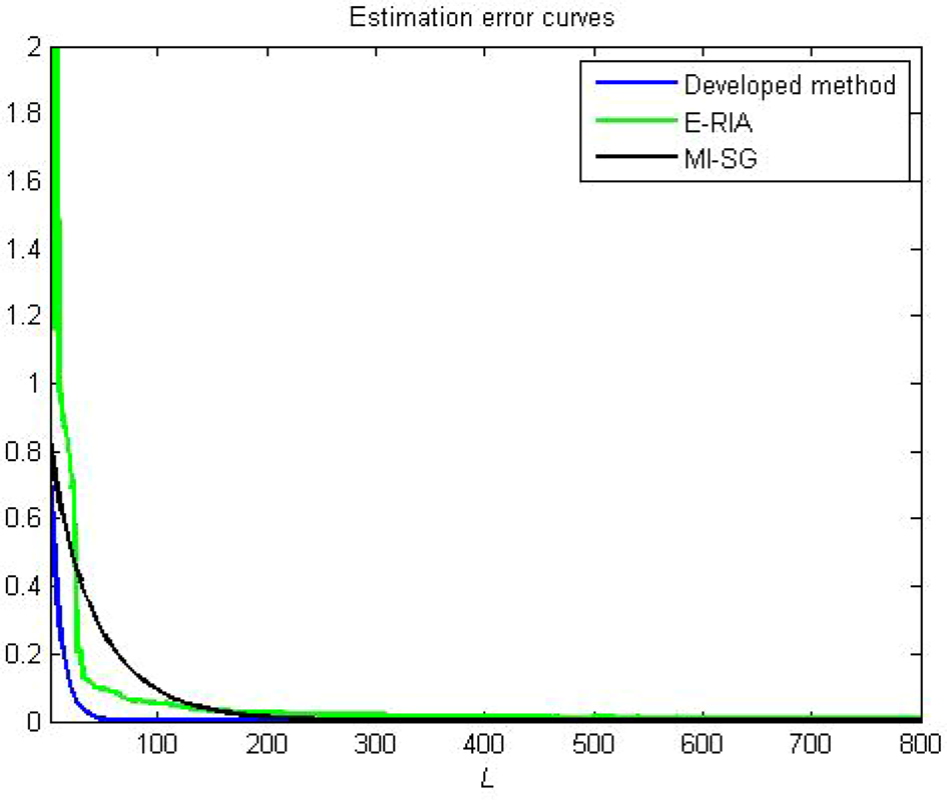
Comparison parameter estimation errors.

Figures 4, 5 and 6 provide the estimation profiles of the parameter identification results obtained by the three estimators. Note that the estimated parameters immediately and sharply tend toward the desired values as the samples are fed into the estimators. Additionally, the estimated values converge to the desired parameters as the data length reaches the preset sample length. It is also intuitive that the parameter estimation performance of the developed method yields better convergence than MI-SG and E-RIA. In Fig. 7, the parameter identification curves are shown, in which when the sample increases, all estimation errors decrease gradually, thereby showing that the three identification methods can realise the system’s parameter estimation. The developed method uses minimal time to approach the real value, and its result can be close to the real value, thereby showing the advantage of the developed algorithm.

**Figure 8:**
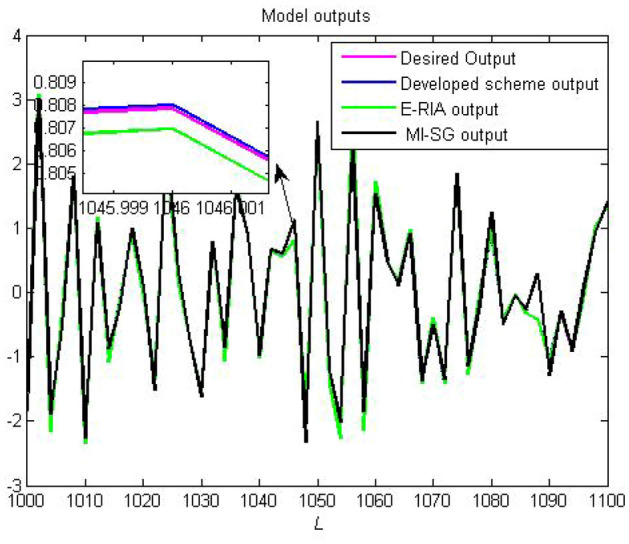
Established model outputs.

**Figure 9:**
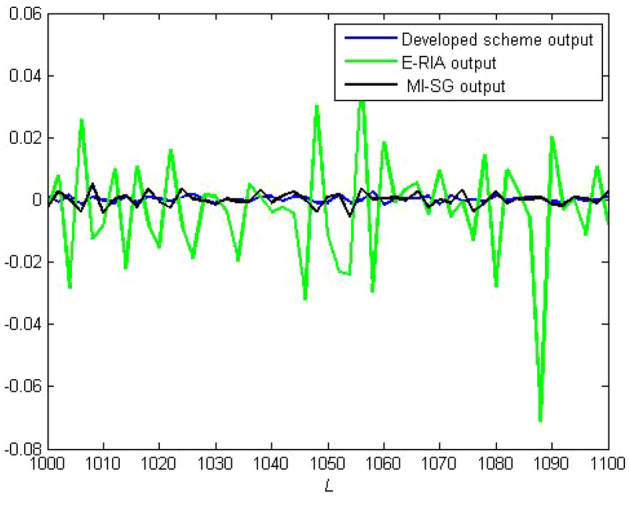
Output errors.

**Figure 10:**
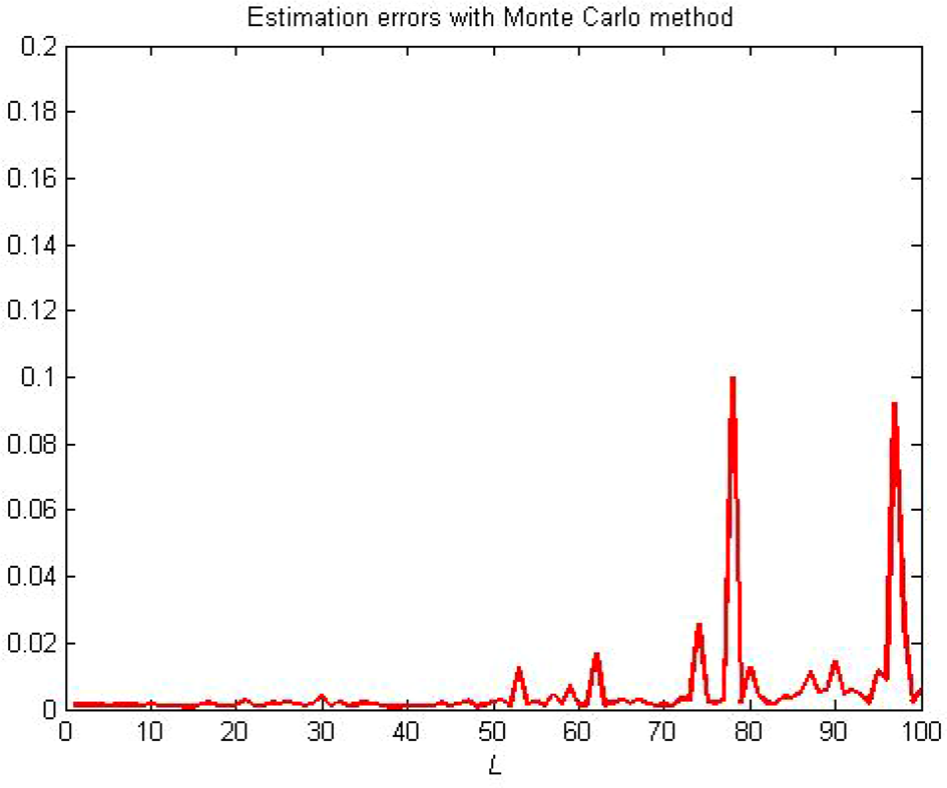
Estimation errors with Monte Carlo method.

One criterion for judging the rationality of an estimation model is to verify whether the estimation model output can effectively track the actual system output performance. The model output and actual system output are presented in Figs. 8 and 9, respectively. Note that the estimation models obtained based on the three estimators can track the real output,thereby demonstrating the effectiveness of MI-SG, E-RIA and the proposed approach. The smallest output error can be obtained by the developed method in comparison to those of MI-SG and E-RIA, in which the superiority of the designed scheme in "Adaptive identification scheme" section is demonstrated. The estimation errors with monte-carlo method are shown in Fig. 10. Note that in 100 independent tests, the estimation error curve fluctuates within a small range without large fluctuations, thereby validating the stability of the proposed method.

### Experiment

**Figure 11:**
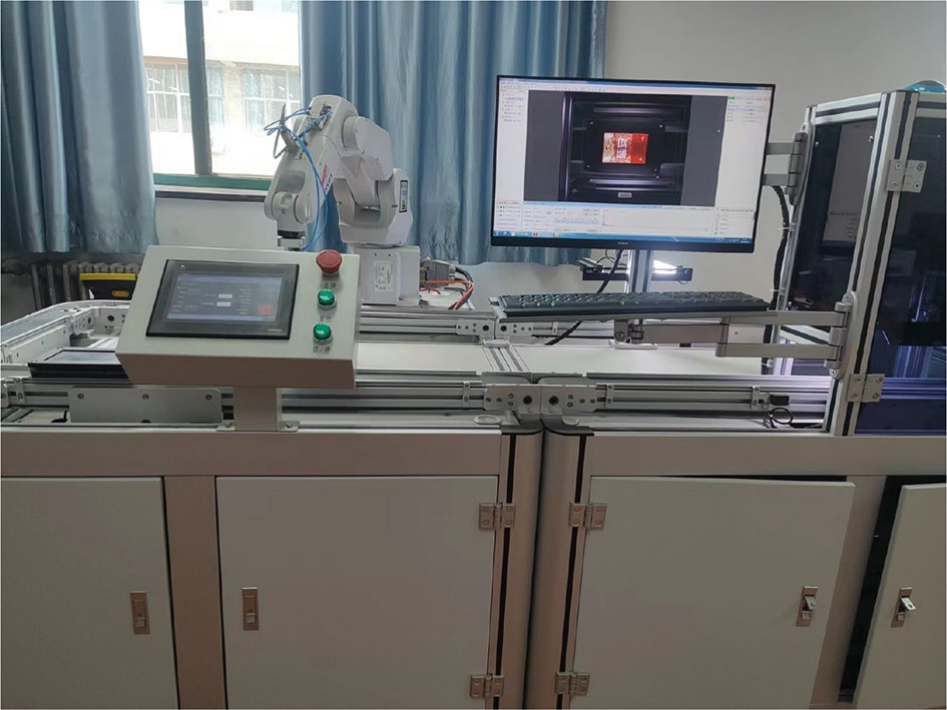
Servo drive system.

As described in Fig. 11, a servo manipulator system is used to test the usefulness of the developed algorithm. A permanent magnet synchronous motor drives the skew-wheel, and which drives the manipulator thereafter to move according to a given trajectory. The platform consists of a permanent magnet synchronous motor (ZLAC60ASM200), a digital signal processing (TMS320F2809), and an encoder (HF154S-A48), etc. The given signal is chosen as $y_{d}=2\sin (1/3\pi t)$.

The system is described as

$$\begin{aligned} \left\{ \begin{array}{lr} \dot{x}_{1}=x_{2}\\ \dot{x}_{2}=\theta _{1}x_{2}+\theta _{2}u-\theta _{3}sign(x_{2})-\theta _{4}x_{2})\\ \end{array} \right. \end{aligned}$$

where $\theta _{1}=\frac{-K_{2}}{J}$,$\theta _{2}=\frac{K_{1}}{J}$, $\theta _{3}=\frac{T_{c}}{J}$, $\theta _{4}=\frac{B}{J}$, $x=[x_{1},x_{2}]^T=[d,\dot{d}]^T$. *d* and $\dot{d}$ represents the angular position and velocity.

**Figure 12:**
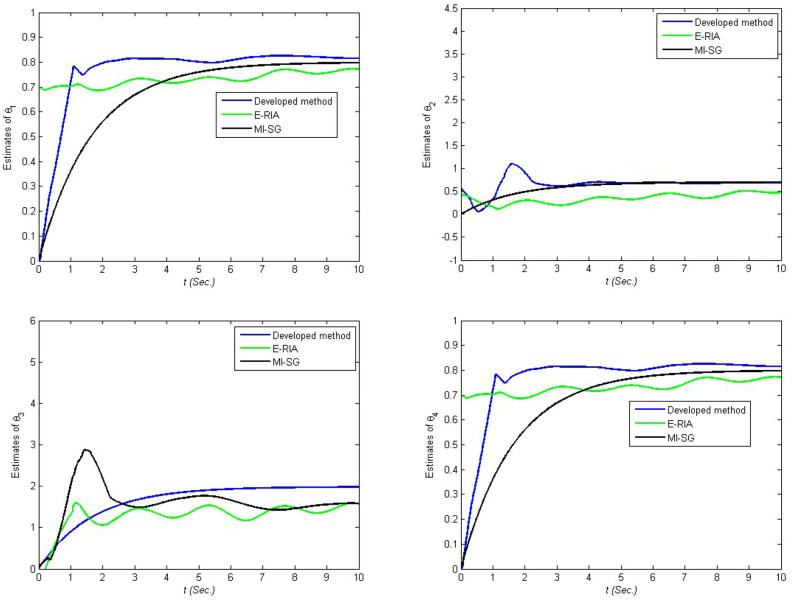
Parameter estimation profiles.

**Figure 13:**
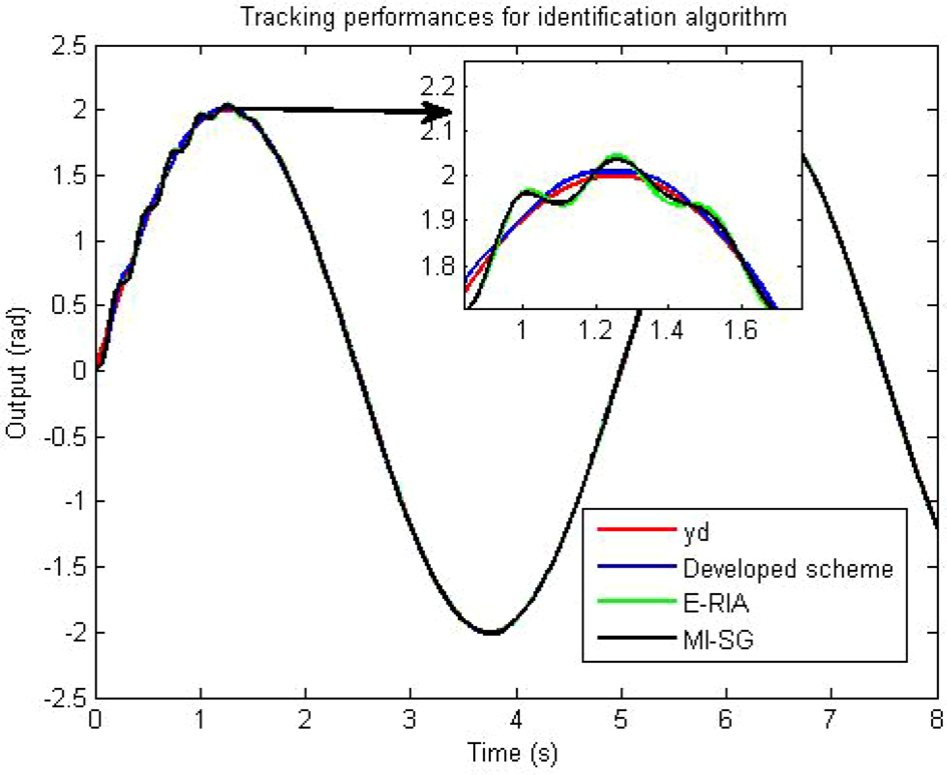
Tracking performance.

**Figure 14:**
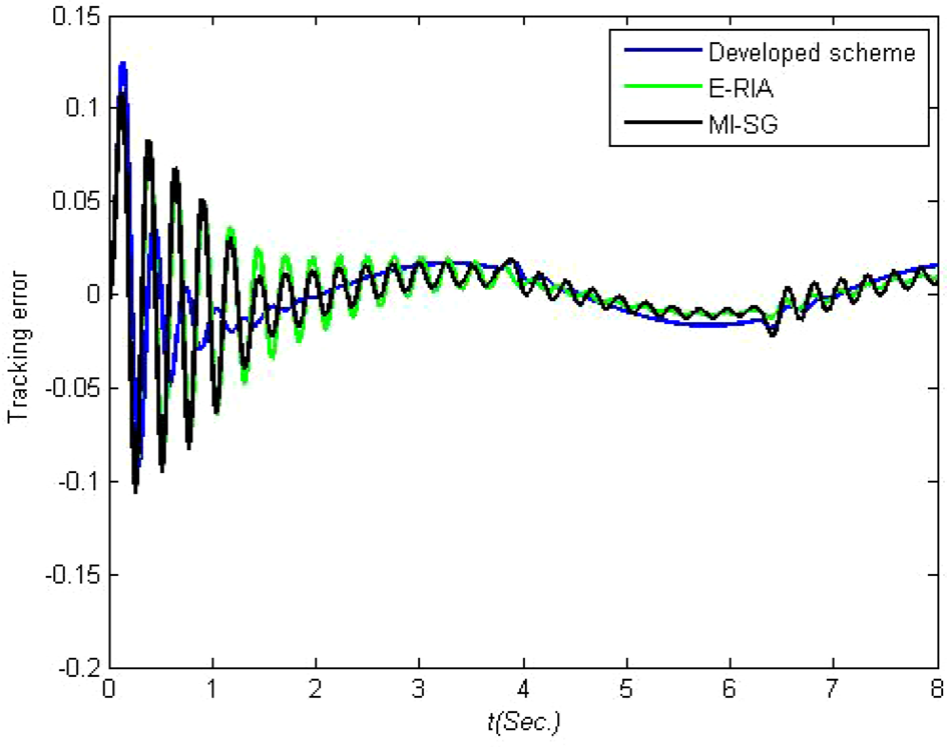
Tracking output error.

The identification results are displayed in Fig.12, in which the estimated parameters fluctuate rapidly in the beginning of the parameter estimation. With increase in time, the estimated parameter curves tend to have stationary values. The developed scheme has fast convergence performance because the proposed algorithm can approach the stationary value in the shortest amount of time. The tracking performance and output error curves are described in Figs. 13 and 14, respectively. The three tested estimation models can represent the dynamics of the actual system output, indicating that MI-SG, E-RIA and the developed approach can effectively identify the parameters of the servo manipulator system. The tracking error results show the advantages of the developed algorithm because of the minimum tracking output error.

Quantitative analysis can further verify the effectiveness of the proposed algorithm. By using the model output error data, some performance indicators are provided.

1. Root Mean Square, $RMS=\sqrt{\frac{1}{L'}\sum _{j=1}^{L'}e(j)^2}$,
2. Prediction Error Mean, $PEM=\frac{1}{L'}\sum _{j=1}^{L'}e(j)$,

where predicted output length is described by $L'$, $e(j)=y(j)-{\hat{y}}(j)$.

Based on the model output error data and performance indicators, the calculated indicator results are listed in Table.1. It can be seen that the indictors provided by the three estimation methods have small values. It indicates that the three considered estimation methods can achieve effective parameter estimation for an actual system. However, the developed algorithm has smaller values than the MI-SG, E-RIA methods, demonstrating excellent identification performance compared with that of the other two estimators.

**Table 1:** Index values.

Algorithm	RMS	PEM
MI-SG	2.1985 × 10^−4^	− 3.0152 × 10^−4^
E-RIA	4.6952 × 10^−4^	8.1586 × 10^−4^
Proposed method	1.0132 × 10^−4^	− 4.2246 × 10^−4^

## Conclusion

This study presents an optional identification structure for an expanded sandwich system using identification error data. This research allows us to use other errors to design adaptive parameter laws instead of prediction or observation errors. System data can be efficiently used based on the developed filter technology and forgetting coefficient, in which the utilization rate of new data in each recursive step is higher than that of old data. The usefulness and effectiveness of the developed algorithm have been demonstrated by using a numerical example and an experiment conducted on a servo manipulator system. In particular, the parameter identification error convergence performance can be shown from a theoretical perspective by using the martingale difference convergence theorem. In future work, we will extend the proposed scheme to the identification of other systems, such as extended Hammerstein-Wiener systems, bilinear systems and linear systems with varying parameter, etc.
